# Pyloric Atresia

**Published:** 2013-01-01

**Authors:** Vivek Gharpure

**Affiliations:** Department of Pediatric Surgery, Children's Surgical Hospital 13, Pushpanagari, Aurangabad, 431001, India

 (This section is meant for residents to check their understanding regarding a particular topic)

## Questions

1. Discuss the epidemiology of pyloric atresia.
2. Describe the etiology of pyloric atresia.
3. What anomalies are associated with pyloric atresia?
4. Discuss the genetics of pyloric atresia.
5. How can pyloric atresia be antenatally diagnosed?
6. What are clinical features of pyloric atresia?
7. What investigations will be necessary in a patient with pyloric atresia?
8. How should a patient with pyloric atresia be handled?
9. What surgical interventions are done in pyloric atresia?
10. How parents of a baby with pyloric atresia should be counseled?

## Answers

Its incidence is approximately 1 in 100,000 newborns and constitutes about 1% of all intestinal atresias [1].
It is not exactly known. Vascular theory and failure of canalization theory have been proposed, but there is inadequate embryological evidence in support of either [1].
Junctional epidermolysis bullosa, ureteral and renal anomalies (dysplastic/multicystic kidney, hydroureteronephrosis, ureterocele, duplicated renal collecting system, and absent bladder), aplasia cutis congenita, milia, nail dystrophy, and multiple colonic atresias [1, 2].
Mutations in ITGB4 (~80%), ITGA6 (~5%), and PLEC1 (~15%) genes cause epidermolysis bullosa and pyloric atresia (EB-PA).
Oligohydramnios on antenatal USG, large gastric bubble, and presence of echogenic material in amniotic fluid.
Non-bilious vomiting, feed intolerance, and upper abdominal distension results from gastric outlet obstruction. Scaling and blistering of skin is found with associated epidermolysis bullosa. Whole of the abdomen can be distended in case of associated colonic atresia on account of accumulation of bile in the bowel between pyloric and distal atresias; however, radiograph depicts only gastric shadow as no air can be passed beyond pyloric atresia [1, 2].
A single gastric air bubble with no gas distally on plain abdominal radiograph gives suspicion of pyloric atresia, however it should be kept in mind that preampullary duodenal obstruction may also sometimes produce single gastric bubble [3, 4]. Air or contrast can be put in the stomach in case of doubt. Clinical diagnosis is unreliable and examination of a skin biopsy is usually required to establish the diagnosis of EB with PA, especially in infants. These patients have skin infections and cultures, septic markers will also be necessary.
Patient must be minimally touched and handled even for vascular access. Adhesive dressing or tapes should never be used. To monitor the oxygen saturation, a probe of the 'clip-on' variety placed on a digit or the ear lobe is ideal.
(i) Mickulicz type Pyloroplasty which involves incising the canal longitudinally, excising membrane, and transverse closure of pylorus. (ii) Longitudinal incision of the atretic pylorus, isolation of the cul-de-sacs of gastric and duodenal mucosa, and end-to-end anastomosis. The longitudinal pyloromyotomy is closed longitudinally [5]. (iii) A side to side gastro-duodenostomy has been described too [6].
This autosomal recessive inherited disease is usually fatal within the first few weeks or months of life even following surgical correction of the intestinal obstruction. Psychosocial support, including social services and psychological counseling is necessary. EB-PA is an autosomal recessive disorder. At conception, each sibling of an affected individual whose parents are both carriers has a 25% chance of being affected, a 50% chance of being an asymptomatic carrier, and a 25% chance of being unaffected and not a carrier [1]. Carrier status of parents needs to be confirmed with molecular genetic testing.

## Footnotes

**Source of Support:** Nil

**Conflict of Interest:** None
